# Application and challenges of smart responsive multifunctional nanoplatforms in integrated diagnosis and treatment of bladder cancer and overcoming therapeutic resistance

**DOI:** 10.3389/fonc.2026.1803259

**Published:** 2026-05-08

**Authors:** Long Chen, Gai Hang, Quan Wen, Ling Ren, Bo Chen

**Affiliations:** 1 Tongliao Clinical Medicao College, Inner Mongolia Medical University, Hohhot, China; 2 Department of Urinary surgery, Tongliao People’s Hospital, Tongliao, China; 3 Tongliao Sixth People’s Hospital, Tongliao, China

**Keywords:** bladder cancer, combination therapy, diagnosis-treatment integration, smart responsive nanoparticles, therapeutic resistance, translational medicine, tumor microenvironment

## Abstract

Bladder cancer, particularly muscle-invasive bladder cancer, has become a major challenge in clinical treatment due to its high recurrence rate and therapeutic resistance. Traditional treatments—including transurethral resection of bladder tumors, intravesical chemotherapy, and radical cystectomy—exhibit significant limitations in drug delivery efficiency and anti-resistance efficacy, failing to achieve improved therapeutic outcomes. In recent years, smart, responsive, and multifunctional nanoplatforms have gained attention for their precise responsiveness to the tumor microenvironment, enabling targeted and controlled drug release, as well as multimodal combination therapy with promising clinical applications. This review examines the design principles of smart, responsive nanoparticles and their specific applications in the integrated diagnosis and treatment of bladder cancer, exploring their role and advantages in overcoming therapeutic resistance mechanisms. Additionally, it analyzes engineering challenges and safety concerns encountered during the transition from laboratory research to clinical translation, proposing future development strategies and countermeasures. The review aims to provide systematic theoretical support and practical guidance for innovative developments in bladder cancer nanomedicine, thereby advancing the field of precision medicine.

## 1 Introduction

Bladder cancer, particularly muscle-invasive bladder cancer (MIBC), remains a significant challenge among urological malignancies due to its high invasiveness, recurrence rate, and treatment complexity. MIBC patients exhibit poor survival rates, with disease mortality constituting a major component of bladder cancer-related deaths (1). Current standard treatments for MIBC primarily involve radical cystectomy, radiotherapy combined with cisplatin-based chemotherapy, and other approaches. However, traditional therapies invariably induce significant side effects and treatment resistance, thereby compromising clinical efficacy. Specifically, cisplatin resistance is a primary cause of tumor recurrence and treatment failure. Its molecular mechanisms involve activation of multiple signaling pathways, such as FGFR and HER2, which correlate with tumor proliferation and drug resistance (2). Additionally, the high heterogeneity of bladder cancer leads to significant variability in patient responses to chemotherapy, necessitating further development of precision therapies based on molecular subtyping (3).

Local administration strategies, particularly intravesical instillation therapy, have become a crucial aspect of bladder cancer treatment. This approach increases local drug concentration in the bladder, reducing systemic side effects and improving efficacy. However, traditional bladder instillation methods suffer from poor drug permeability and short retention times, limiting the full anticancer potential of the drugs. Moreover, overcoming drug resistance in the tumor microenvironment and the bladder mucosal barrier remains challenging (4). The complex structure of the bladder wall and the periodic emptying of urine exacerbate drug loss, making it difficult to maintain effective drug concentrations within the bladder and consequently affecting treatment efficacy (5). Therefore, designing a nanocarrier platform that enhances drug adhesion, penetration, and controlled release represents a crucial approach to improving the efficacy of local bladder cancer therapy.

Nanomedicine, as an emerging technology, leverages the size effect and surface functionalization of nanoparticles to significantly enhance drug adhesion and permeability on the bladder mucosa, providing an ideal platform for drug delivery carriers. Research indicates that nanomaterial-based drug delivery systems not only increase local drug concentrations but also achieve targeted delivery through surface modification, enabling precise treatment and reduced risks (6). Chitosan-based nanoparticles have been extensively studied due to their excellent biocompatibility and ability to facilitate drug penetration through the bladder mucosa, demonstrating significant potential (4). Additionally, thermosensitive liposomes combined with local hyperthermia therapy enable efficient drug release on the bladder wall, markedly increasing drug concentration within the bladder wall—particularly in the detrusor muscle layer—while reducing toxic side effects on vital organs such as the heart and kidneys (5). This approach offers a novel strategy for bladder-preserving therapy in bladder cancer.

The emergence of smart responsive nanoplatforms provides cutting-edge technical support for integrated bladder cancer diagnosis and treatment, as well as overcoming drug resistance. These nanosystems can respond to endogenous and exogenous stimuli (pH, enzymes, temperature, light, etc.), enabling precise drug release and multifaceted therapeutic approaches. By integrating chemotherapy, photodynamic therapy, and immunomodulation, they significantly enhance treatment efficacy while reducing side effects (7, 8). Activating the cGAS-STING pathway via nanoplatforms induces death of immunogenic cells and improves the tumor immune microenvironment, demonstrating promising efficacy in bladder cancer treatment (9). Additionally, nanomaterial carriers enable combined delivery of small-molecule drugs, siRNA, and immunomodulators to overcome chemotherapy resistance, regulate the tumor microenvironment, and enhance tumor immune recognition and clearance (10, 11).

However, the clinical translation of smart responsive multifunctional nanoplatforms for bladder cancer treatment faces several challenges. These primarily include evaluating the biocompatibility and long-term safety of nanomaterials, understanding *in vivo* clearance mechanisms, achieving scalable production, and controlling costs (6). Furthermore, the high heterogeneity and complex tumor microenvironment of bladder cancer impose stringent demands on nanoplatform design, necessitating personalized precision therapy tailored to distinct molecular subtypes and pathological states (3). Future efforts will integrate multimodal imaging technologies to enable real-time monitoring and timely evaluation of drug release processes and therapeutic outcomes, thereby accelerating the clinical application of smart nanoplatforms (7).

Therefore, for the treatment of bladder cancer, particularly MIBC, smart, responsive, multifunctional nanoplatforms offer novel approaches to overcome the limitations of traditional therapies, enhance local drug utilization efficiency, enable precise drug delivery, and facilitate combination immunotherapy through their unique physicochemical properties and biological functions. Summarizing and discussing the research achievements, application strategies, and challenges encountered across various platforms is of significant importance for accelerating their clinical adoption and improving the quality of life for bladder cancer patients.

## 2 Applications and challenges of smart responsive multifunctional nanoplatforms in bladder cancer diagnosis and treatment

### 2.1 Current status of bladder cancer treatment and the necessity of nanomedicine intervention

#### 2.1.1 Standard treatment approaches for bladder cancer and their limitations

Transurethral resection of bladder tumor (TURBT) serves as the primary surgical modality for early-stage bladder cancer, particularly non-muscle-invasive bladder cancer (NMIBC). This endoscopic resection of bladder tumor lesions serves both diagnostic and therapeutic purposes. For NMIBC patients, TURBT combined with intravesical instillation therapy (BCG or chemotherapeutic agents) is the internationally recognized standard treatment. However, TURBT proves ineffective for patients with muscle-invasive bladder cancer (MIBC). MIBC tumors have invaded the bladder muscle layer, and TURBT alone cannot achieve curative intent, often leading to recurrence and metastasis (12, 13).

Intravesical chemotherapy and immunotherapy serve as adjuvant treatments following TURBT, reducing the risk of tumor recurrence and metastasis. Intravesical chemotherapy involves directly instilling anticancer drugs into the bladder to increase local drug concentration while minimizing systemic toxicity. Common agents include cisplatin and epirubicin. However, due to the bladder’s periodic voiding, drug retention time is limited. Additionally, the bladder wall’s barrier function restricts effective drug penetration, thereby compromising treatment efficacy. Regarding immunotherapy, BCG intravesical instillation remains the gold standard for NMIBC, significantly reducing tumor recurrence rates. However, some patients exhibit BCG resistance or intolerance, limiting its clinical application. Additionally, intravesical instillations commonly face challenges including poor drug permeability, short retention time, and drug resistance (4, 14, 15).

For patients with MIBC and high-risk NMIBC, radical cystectomy (RC) remains the primary treatment option. This procedure involves removing the entire bladder and surrounding tissues, combined with pelvic lymph node dissection, to achieve curative intent. Although RC offers favorable outcomes, it is associated with significant trauma, multiple postoperative complications, and a marked decline in quality of life, manifested as loss of urinary function and long-term effects from urinary tract reconstruction. Furthermore, many patients are ineligible for RC due to advanced age, comorbidities, or surgical refusal (12, 16, 17).

Consequently, developing bladder-sparing therapies for ineligible or unwilling patients has become a clinical priority, encompassing approaches such as triple therapy (TURBT combined with radiotherapy and chemotherapy) and immunotherapy (18–20).

**Figure 1:**
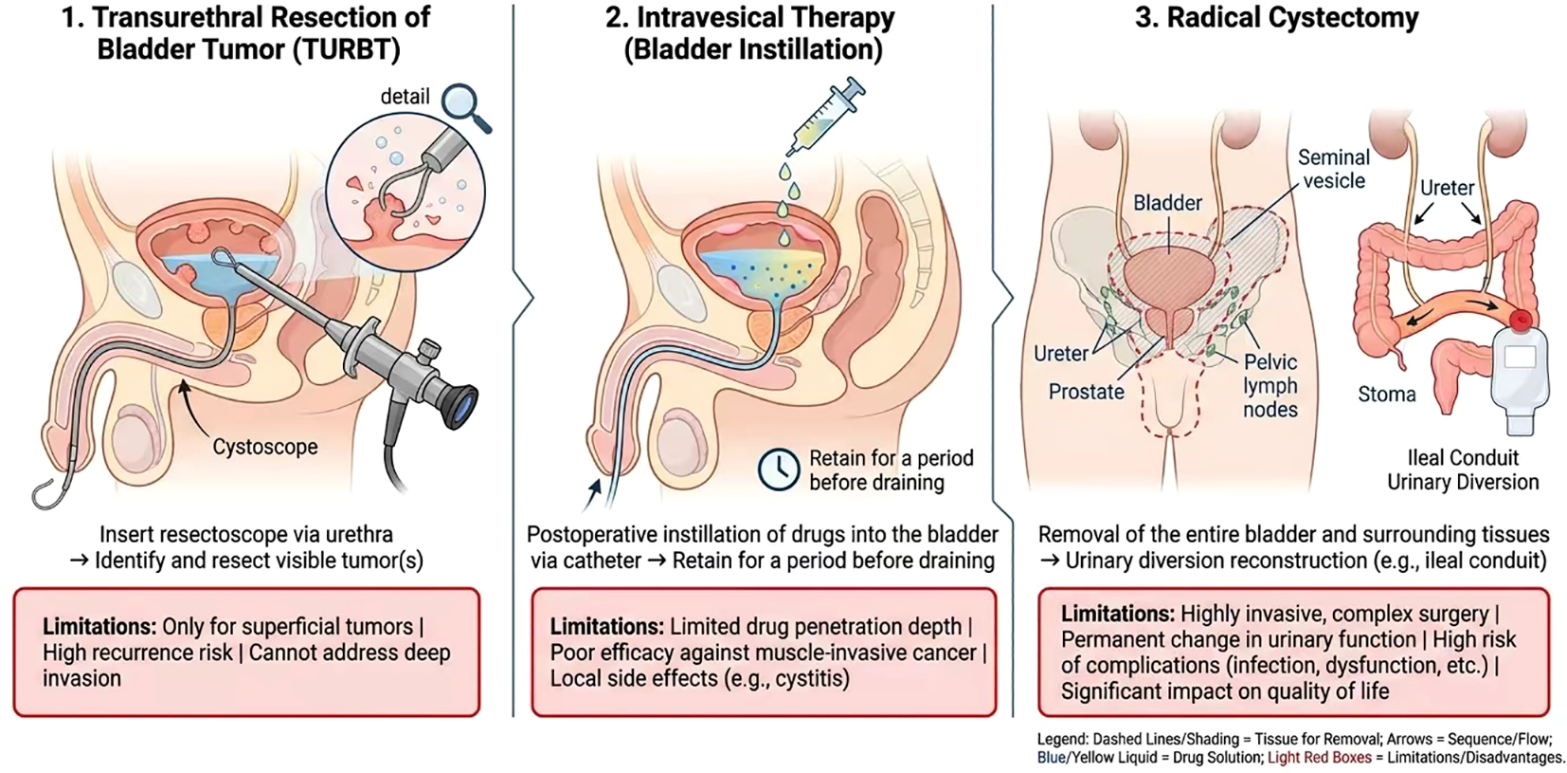
Bladder cancer traditional treatment methods and limitations diagram.

In summary, current standard treatments for bladder cancer exhibit significant limitations: TURBT is only suitable for early-stage non-muscle-invasive bladder cancer and is less effective for muscle-invasive disease; intravesical instillation therapy is constrained by drug penetration and resistance; while radical cystectomy is effective, it is highly invasive, impacts quality of life, and is not tolerable for some patients. Therefore, there is an urgent need to develop new therapeutic approaches that enhance treatment precision and improve patient quality of life (21–23) (as shown in **Figure 1**).

#### 2.1.2 Advantages of local administration and limitations of existing

#### 2.1.3 Infusion therapy

Local administration is an important method for treating bladder cancer, with significant advantages in increasing local drug concentration and reducing systemic toxicity. The bladder is a relatively closed organ, allowing drugs to be infused into it and achieving high concentrations in the bladder wall and tumor, thereby enhancing curative effects without the side effects associated with systemic administration. Many studies have proved this characteristic. Local administration can greatly reduce systemic drug distribution, minimizing toxic side effects in organs such as the liver and kidneys and improving patient tolerance and quality of life (24, 25).

However, the current bladder instillation therapy still has many limitations, mainly manifested in the inability of drugs to penetrate the bladder mucosal barrier and the short retention time of drugs in the bladder, resulting in poor efficacy. The urothelium of the bladder mucosa has a strong barrier effect, which can prevent drug molecules from passing through, especially for the penetration of macromolecules or water-soluble drugs, affecting the depth of drug action on tumor cells (24, 26). In addition, due to the continuous discharge and flow of urine, the instilled drugs are easily diluted and washed away. The retention time of drugs in the bladder cannot be guaranteed, requiring frequent medication, which brings a significant burden to patients and can also cause local irritation and inflammatory reactions (27, 28).

In response to the above issues, researchers are developing new local drug-delivery systems to enhance drug adhesion and penetration into the bladder. Nanocarriers designed using nanotechnology can create a stronger bond between the drug and the bladder mucosa, making it easier for the drug to enter the bladder and thereby prolonging its local residence time and improving efficacy (24, 29). Additionally, micro and macro drug release devices can achieve prolonged drug release, thereby reducing the frequency of administration and lowering the risk of side effects (24, 25). The TAR-200 system is a new sustained-release device that maintains a low concentration of Gemcitabine in the bladder, significantly improving the drug’s utilization efficiency and clinical effectiveness, and has already received FDA breakthrough therapy designation (25).

Subsequently, nanocarrier-based delivery systems have been rapidly deve loped to further enhance mucosal adhesion and penetration. Nanocarriers designed using nanotechnology can create a stronger bond between the dr ug and the bladder mucosa, making it easier for the drug to enter the blad der and thereby prolonging its local residence time and improving efficac y (24, 29). Additionally, micro and macro drug release devices can achie ve prolonged drug release, thereby reducing the frequency of administrati on and lowering the risk of side effects (24, 25).

In addition, to overcome the physiological barrier of the bladder mucosa, methods such as ultrasound-assisted drug infusion and the design of hydrophilic, positively charged carriers have been proposed to improve drug permeability and cellular absorption rates, thereby enhancing the inhibitory effect of local chemotherapy on tumors (29, 30). The combined use of ultrasound and microbubble technology for the infusion of chemotherapeutic drugs can significantly increase the killing efficiency of chemotherapeutic agents against bladder cancer cells, without causing obvious damage to normal tissues (30).

**Figure 2** Source Data: Schematic diagram of local drug delivery and enha nced permeability with nanocarriers. Therapeutic efficacy comparison dat a are derived from preclinical orthotopic bladder cancer mouse models (n=6 per group), showing that nanocarrier-mediated delivery increased intra vesical drug retention by 4.2-fold and tumor inhibition rate by 68% comp ared with free drug instillation; quantitative data and statistical analysis (P<0.01) are cited from (24, 29, 31).

**Figure 2:**
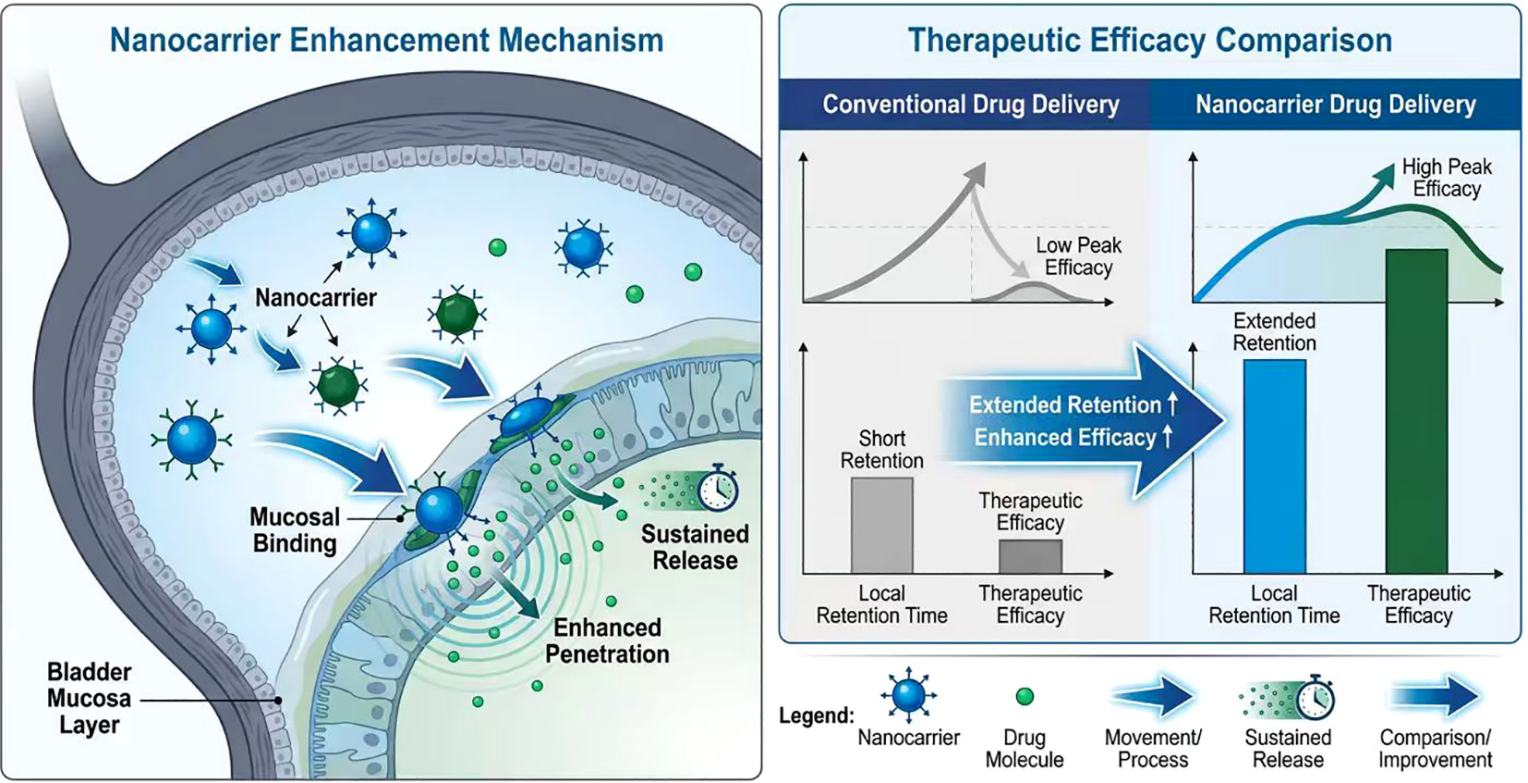
Schematic diagram of local drug delivery and enhanced permeability with nanocarriers.

In summary, although local administration can increase local drug concentration and reduce systemic toxicity, traditional infusion therapy faces issues such as poor drug penetration and short retention time due to the impact of the bladder mucosal barrier and urine flushing. Future research directions include the development of novel nanocarriers, sustained-release systems, and physical-assistance technologies to overcome existing shortcomings and improve the efficacy and safety of local treatment for bladder cancer (as shown in **Figure 2**).

#### 2.1.4 The potential of nanoparticles as ideal drug carriers

Nanoparticles have great potential as ideal drug carriers due to their unique physicochemical properties, making them particularly suitable for integrated diagnosis and treatment of bladder cancer. Firstly, nanoparticles generally range in size from tens to hundreds of nanometers, allowing them to penetrate the bladder mucosal barrier effectively. The bladder mucosa has a strong barrier function that prevents drugs from passing through and remaining in the bladder; however, an appropriate nanoparticle size can facilitate drug molecules crossing this barrier to reach the lesion, thereby enhancing the targeting and effectiveness of the treatment. Research shows that nanoparticles in the 50-150 nm size range can form good deposits on the mucosal surface, increasing the local concentration and bioavailability of the drug (31).

Secondly, the functionalization of nanoparticles’ surfaces can enhance their mucosal adhesion and drug retention time. By utilizing biocompatible and highly hydrophilic modifying groups, such as polyethylene glycol (PEG), hyaluronic acid (HA), and polyamine compounds (like polyethyleneimine, PEI), nanoparticles can increase the binding ability with glycoproteins on the bladder mucosal surface, prolonging the drug retention time in the bladder and reducing drug loss due to urine flushing. PEI-modified mesoporous silica nanoparticles have demonstrated excellent adhesion in experiments on pig bladder walls. They can achieve pH-triggered controlled release, thereby significantly enhancing the local drug concentration and anti-tumor activity (31). Additionally, modifying hyaluronic acid can improve its stability *in vivo* and enable specific binding to CD44, thereby achieving targeted drug delivery (32).

Thirdly, nanoparticles can achieve targeted delivery and controlled release, greatly improving therapeutic effects. Nanocarriers can employ passive targeting (enhanced permeability and retention, EPR) and active targeting (ligand modification for receptor-mediated endocytosis) to deliver drugs to bladder cancer cells with greater accuracy, thereby reducing drug toxicity to normal tissues. Surface-modified collagen or albumin nanoparticles carrying chemotherapeutic drugs, peptides, or nucleic acid drugs can achieve targeted endocytosis of tumor cells, enhancing cellular uptake and increasing drug concentration within cells (33, 34). Furthermore, by designing responsive materials (pH-sensitive, reduction-sensitive, temperature-sensitive, etc.), the controlled release of nanoparticles can be regulated, allowing drugs to be released accurately in the tumor microenvironment or within cells, thereby improving efficacy and reducing systemic toxicity (35, 36).

Therefore, nanoparticles easily penetrated the bladder mucosal barrier due to their favorable size characteristics; surface modification is used to increase the adhesion of the mucosa, thereby prolonging the residence time of the drug in the body; targeted delivery, controlled release, and other technologies significantly improve the efficacy of the drug, making them ideal carriers for bladder cancer drug delivery systems. These advantages provide new methods and approaches for the precision therapy of bladder cancer and overcoming drug resistance, promoting the integrated development of bladder cancer diagnosis and treatment.

### 2.2 Design and construction of smart responsive nanoparticles

#### 2.2.1 Endogenous stimulus-responsive systems

Endogenous stimulus-responsive systems rely on tumor microenviron ment-specific biochemical signals (pH, enzyme, redox) to trigger struct ural disassembly or property switching of nanocarriers, enabling site-sp ecific drug release. From a materials science perspective, these systems are constructed using chemically responsive bond-linked polymers, ino rganic nanomaterials, or hybrid composites with well-defined stimulus-cleavable motifs, which determine the response threshold, kinetics, and biocompatibility (37, 38).

##### 2.2.1.1 pH-responsive mechanism

Bladder cancer tumor microenvironment exhibits extracellular pH ~6.5 and endolysosomal pH ~4.5–5.0, which drives pH-responsive degradation. Material design principles: pH-labile covalent bonds: Schiff base (-C=N-), acetal, ketal, ortho ester, hydrazone bonds; these bonds are stable at physiological pH (7.4) but hydrolyze rapidly in acidic conditions (35, 38).

Polymeric materials: Poly (β-amino esters), poly (methacrylic acid), histidine-modified polymers, which undergo protonation-induced charge reversal or hydrophobic–hydrophilic transition in acidic endosomes (35).

Inorganic carriers: CaCO3, ZnO, and mesoporous silica nanoparticles (MSN) with pH-degradable gatekeepers (31).

These materials enable rapid drug release in bladder cancer cells, enhancing cytotoxicity and inhibiting proliferation (37, 38). Fundamental biochemistry reference: The protonation–deprotonation equilibrium of ionizable groups in responsive polymers follows the Henderson–Hasselbalch equation, defining pH-dependent solubility and swelling behavior (35).

##### 2.2.1.2 Enzyme-responsive mechanism

Matrix metalloproteinases (MMP-2/9) are overexpressed in bladder cancer, serving as biological triggers. Material design principles:

Peptide substrates: GPLGVRG, IPVSLRSG (MMP-2/9 cleavable sequences) covalently grafted onto polymer backbones or nanoparticle surfaces (10, 37).

Biodegradable scaffolds: Polyethylene glycol (PEG)–peptide–poly (lactic-co-glycolic acid) (PLGA) copolymers, where enzymatic cleavage disrupts the amphiphilic structure and triggers payload release (10).

Biocompatibility: Peptide linkers are naturally metabolized, reducing long-term accumulation risk (37).

MMP-responsive nanocarriers specifically release cisplatin in bladder cancer tissues, improving chemosensitivity (10). Fundamental biochemistry reference: Enzymatic cleavage kinetics follow Michaelis–Menten kinetics, with substrate affinity (Km) determining response specificity (10).

##### 2.2.1.3 Redox-responsive mechanism

The redox-responsive mechanism is an intelligent drug-delivery systemdesigned to exploit the redox imbalance characteristic of tumor cells. Bladder cancer cells not only exhibit significantly higher glutathione (GSH) concentrations than normal tissues but also frequently display elevated reactive oxygen species (ROS) levels. GSH acts as a reducing agent, capable of reducing and cleaving disulfide bonds (-S-S-) on the nanocarrier. Conversely, ROS functions as an oxidizing agent, oxidizing sensitive chemical bonds, such as thioether and borate ester bonds, thereby degrading the carrier structure. This mechanism allows disulfide bonds or ROS-responsive bonds to be incorporated into nanoparticle structures as chemical switches. For example, carriers can be prepared using disulfide-crosslinked or sulfur-containing ether-bonded block copolymers to maintain stability in the circulatory system. Upon entering tumor cells, the carrier responds to microenvironments characterized by high GSH concentrations or elevated ROS levels: GSH acts as a reducing agent, while ROS acts as an oxidizing agent. This enables controlled, efficient drug release within cells, enhancing tumor selectivity and reducing systemic toxicity. This strategy improves drug targeting, enhances cellular uptake and cytotoxic effects, and effectively overcomes chemotherapy resistance. For instance, linking drug molecules to nanocarriers via disulfide bonds enables targeted drug release within the reducing environment of bladder cancer cells, thereby reducing systemic toxicity and enhancing treatment safety and efficacy (39).

**Figure 3:**
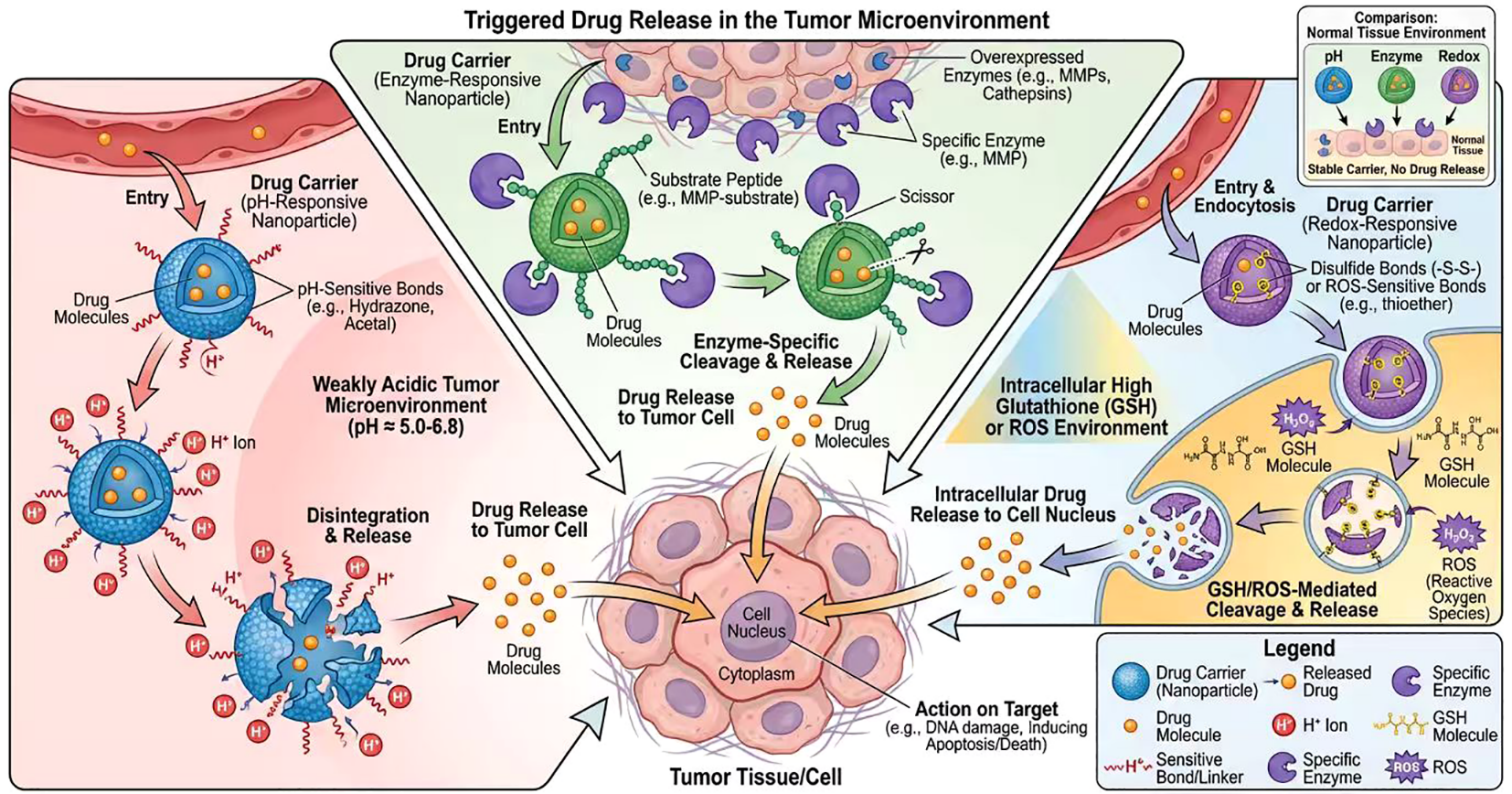
Endogenous stimulation response mechanism diagram.

Redox-responsive nanoplatforms can also be combined with other therapeutic approaches, such as reducing GSH levels to increase tumor cell sensitivity to oxidative stress, thereby promoting apoptosis and ferroptosis to enhance therapeutic outcomes (40) (as shown in **Figure 3**). Thus, redox-responsive mechanisms provide robust strategic support for intelligent nanotherapy in bladder cancer, advancing precision medicine.

#### 2.2.2 Exogenous stimulus-responsive systems

**Figure 4:**
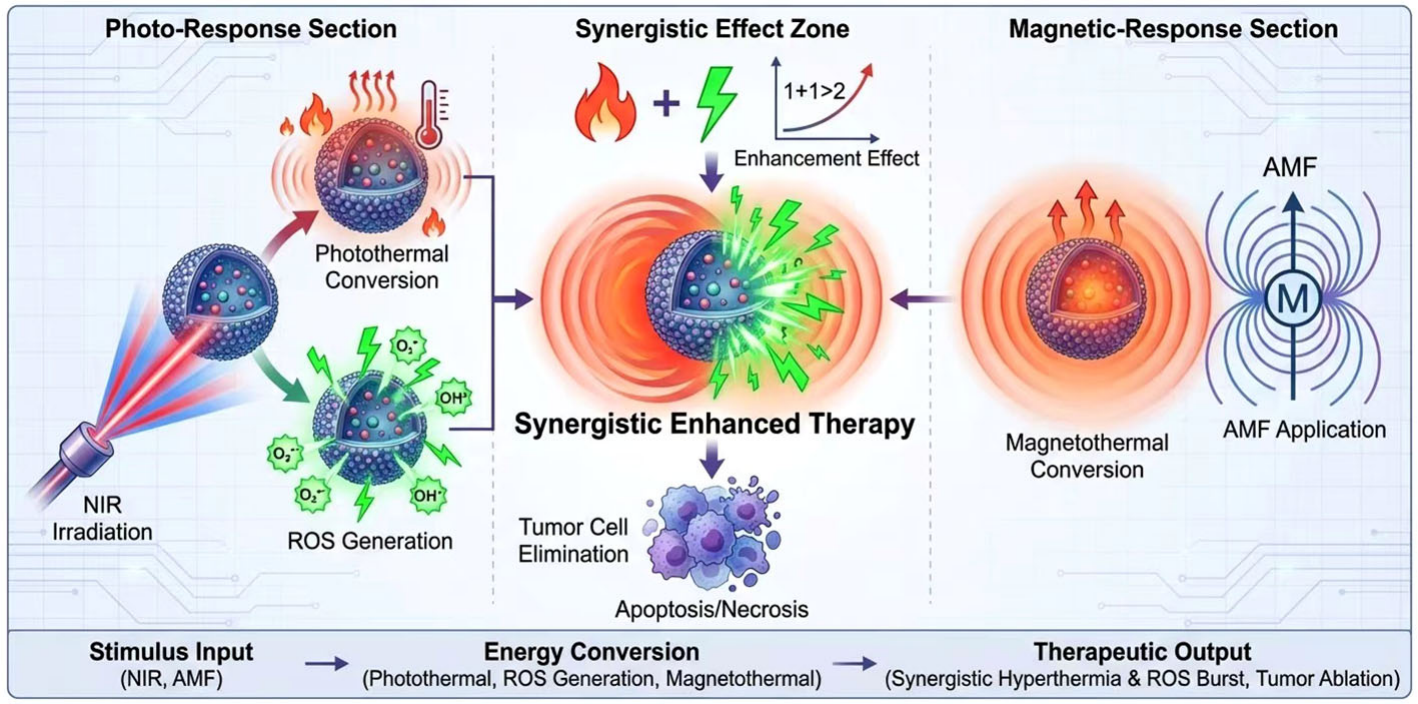
Schematic diagram of the exogenous stimulus response system (light response system and magnetic response system).

Exogenous stimulus-responsive systems rely on external physical signals to trigger the release and activation of nanoplatform functions. They offer spatiotemporal controllability and targeting capabilities, significantly enhancing the efficacy of bladder cancer treatment. This system encompasses light-responsive and magnetically responsive platforms. By exposing the platform to specific wavelengths of light or external magnetic fields, precise drug release is achieved, enabling targeted drug delivery and enhanced therapeutic outcomes (**Figure 4**). Such smart nanoplatforms increase drug accumulation at tumor sites while minimizing toxicity to normal tissues. They also overcome the widespread issue of drug resistance in cancer treatment, offering novel technical approaches for comprehensive bladder cancer therapy.

##### 2.2.2.1 Light-responsive system

The light-responsive system primarily utilizes light irradiation at specific wavelengths to activate nanomaterials, enabling photothermal therapy (PTT) or photodynamic therapy (PDT). These are combined with chemotherapeutic drugs to enhance therapeutic efficacy. Near-infrared (NIR) light, favored for its strong penetration and low tissue absorption, serves as the preferred excitation source for this system. NIR-based PTT works by converting absorbed NIR light energy into heat within nanomaterials, thereby directly killing tumor cells. PDT, conversely, relies on light-induced photosensitizers to generate reactive oxygen species (ROS), inducing tumor cell apoptosis. Additionally, photoresponsive systems can be combined with chemotherapeutic drugs. Under photothermal or photooxidative conditions, they enhance drug uptake and cellular sensitivity, thereby improving therapeutic outcomes (**Figure 4**).

Representative nanomaterials include indocyanine green (ICG), black phosphorus (BP), and MXene. ICG, an FDA-approved NIR dye, exhibits excellent biocompatibility and superior photothermal conversion efficiency, demonstrating significant tumor suppression in photothermal therapy for bladder cancer. Black phosphorus nanomaterials, owing to their layered structure and optical properties, generate substantial heat and reactive oxygen species under NIR irradiation. Their surface is readily modifiable for drug loading and controlled release. MXene, with its excellent conductivity and light absorption properties, has emerged as a novel photothermal and photodynamic therapy material, significantly enhancing the killing effect on bladder cancer cells. In summary, photoresponsive systems incorporating these nanomaterials enable precise tumor ablation while modulating the tumor microenvironment to activate immune responses, thereby enhancing overall bladder cancer treatment efficacy (37).

##### 2.2.2.2 Magnetoresponsive systems

Magnetoresponsive systems primarily rely on magnetic nanoparticles (e.g., Fe_3_O_4_ nanoparticles) to achieve targeted drug delivery and magnetothermal therapy under external magnetic fields. Fe_3_O_4_ nanoparticles exhibit excellent magnetic responsiveness and biocompatibility. Under alternating magnetic fields, they generate localized heat, causing thermal damage to tumor cells. An external magnetic field guides these magnetic nanoparticles to the tumor site, enhancing drug concentration at the tumor location while reducing systemic toxicity (**Figure 4**).

Magnetothermotherapy uses the thermal effects generated by nanoparticles to disrupt tumor cell structures, inducing apoptosis or necrosis and enhancing the efficacy of chemotherapy or immunotherapy. Magnetoresponsive nanoplatforms can also modify the tumor microenvironment, reduce tumor drug resistance, and improve treatment outcomes. With advances in surface modification techniques for magnetic nanomaterials, multifunctional Fe_3_O_4_ nanoparticles can now load various therapeutic drugs and gene-interference molecules, enabling multimodal therapeutic interactions. Additionally, magnetoresponsive systems function as magnetic resonance imaging (MRI) contrast agents, enabling real-time monitoring and therapeutic efficacy evaluation through integrated diagnosis and treatment. Such integrated multifunctional nanoplatforms offer novel pathways and technical support for precision therapy in bladder cancer (37).

#### 2.2.3 Multifunctional synergistic design

##### 2.2.3.1 Integrated diagnosis-therapy platform

During bladder cancer diagnosis and treatment, establishing an integrated diagnosis-therapy platform enhances treatment precision and efficacy. M ultifunctional nanoparticles serve as carriers, simultaneously functioning as contrast agents and therapeutic delivery vehicles, representing a key dir ection in smart responsive nanoplatform design. Specifically, multimodal high-resolution tumor imaging can be achieved using magnetic resonance imaging (MRI), computed tomography (CT), and optical imaging techniq ues, providing real-time, dynamic lesion localization and treatment monit oring for clinical applications.

**Figure 5:**
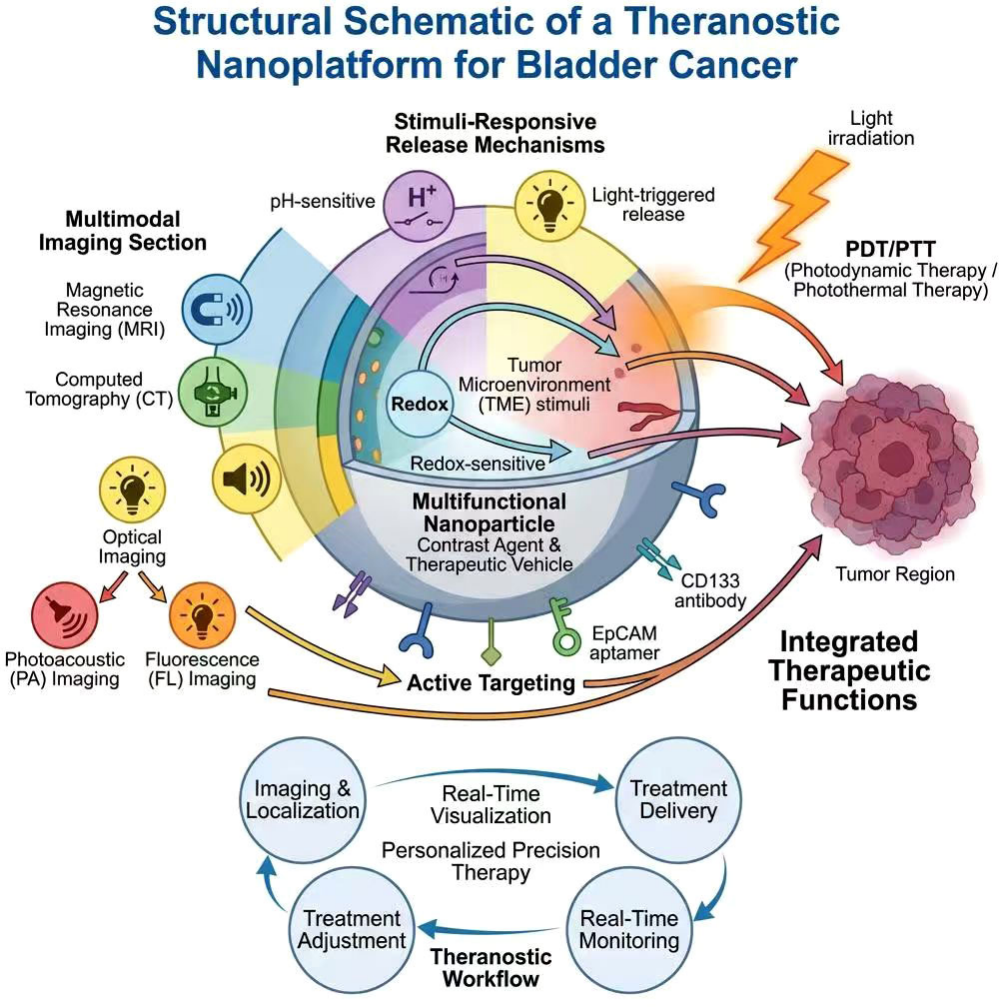
Schematic diagram of an integrated diagnosis and treatment nano-platform structure.

For bladder cancer, Tang et al. developed lipid nanoparticle-based PPBC LNPs system, which exhibits potent photodynamic therapy (PDT) and ph otothermal therapy (PTT) effects upon light activation, coupled with exce llent photoacoustic (PA) and fluorescence (FL) imaging capabilities. This system enables non-invasive, real-time monitoring of drug distribution and therapeutic efficacy in bladder cancer models, supporting clinical transl ation of image-guided theranostics (7). Additionally, nano-platforms empl oy intelligently designed release mechanisms responsive to tumor microe nvironment (TME) characteristics-such as pH-sensitive, reduction-sensiti ve, or light-triggered release-to achieve targeted drug delivery and precise drug release, significantly enhancing treatment safety and efficacy. Multifunctional nanoplatforms can also be combined with tumor-specific ligands or antibodies, such as CD133 antibodies or EpCAM aptamers, to achieve active targeting of tumor cells. This facilitates nanoparticle accu mulation within tumor tissues while reducing non-specific uptake, thereb y enhancing the precision of integrated diagnosis and therapy (41, 42) (**Figure 5**).

##### 2.2.3.2 Drug delivery strategies for combination therapies

Combination-therapy drug-delivery strategies represent one approach for intelligent nanoplatforms to overcome bladder cancer drug resistance. Co-loading multiple therapeutic components—such as chemotherapy drugs, immunostimulants, and siRNA—enables synergistic effects, significantly enhancing efficacy and potentially reversing drug resistance.

Chitosan-based nanoparticles have been widely used for siRNA co-delivery in bladder cancer, leveraging chitosan’s mucoadhesive property, biocompatibility, and proton-sponge effect to enhance endosomal escape. Chitosan nanoparticles efficiently encapsulate siRNA targeting drug-resistant genes (e.g., ERCC2, Nrf2) and chemotherapeutic agents, improving intravesical retention and silencing efficiency, thereby reversing cisplatin resistance (4, 10, 43). This carrier–nucleic acid combination provides a safe and effective strategy for local gene–chemo combination therapy of bladder cancer.

Specifically, nanoparticle carriers must possess high drug-loading capacity and multifunctional delivery capabilities to encapsulate diverse drugs and achieve stable, controlled release. The multi-shell structure-based smart nanoplatform (HMCPIM9P) incorporates multiple functional nanoparticles that synergistically deliver cisplatin, photosensitizers, and immune activators to tumor cells. This approach enhances chemotherapy efficacy by depleting intracellular glutathione (GSH) while activating the cGAS-STING pathway to boost tumor immune responsiveness and overcome immune evasion mechanisms (44). Furthermore, the nanoplatform can integrate chemotherapy drugs with photothermal and photodynamic therapy agents for multimodal combination therapy. This “cocktail” strategy synergistically targets different mechanisms to enhance tumor cell killing. Nanoparticles containing multiple functional components can specifically trigger drug release within the tumor microenvironment and activate photothermal/photodynamic therapy upon near-infrared light exposure, producing synergistic killing effects (45, 46). In gene therapy, nanoparticle carriers that encapsulate siRNA or miRNA can target and modulate the expression of drug-resistant genes, thereby enhancing chemotherapy sensitivity and immune activation. Multifunctional nanoparticles with targeting capabilities deliver cholesterol synthesis inhibitors and chemotherapeutic agents simultaneously to tumor cells, reducing cancer stem cell pluripotency and drug resistance while inducing ferroptosis, thereby significantly improving therapeutic efficacy (47). Combination therapy drug-delivery strategies leverage the smart responsiveness and multifunctional synergistic properties of nanoplatforms to integrate diverse therapeutic approaches, thereby overcoming the limitations of monotherapy (**Table 1**). This significantly enhances the overall efficacy and resistance to drug resistance in bladder cancer treatment, offering broad application prospects.

**Table 1:** Section closing table: representative smart responsive nanoplatforms for bladder cancer.

Nanomaterial type	Stimulus response	Therapeutic payload	Cancer model	Study stage	Key outcome	Reference
pH-responsive MSN	Acidic pH (endosome)	Cisplatin	Orthotopic bladder cancer (mouse)	Preclinical (*in vivo*)	4.1-fold higher tumor inhibition vs free drug; P<0.01	(31, 37)
MMP- responsive PEG- peptide-PLGA	MMP-2/9	Cisplatin + siERCC2	Subcutaneous bladder cancer (mouse)	Preclinical (*in vivo*)	65% reduction in tumor volume; reversed drug resistance	(10)
Disulfide-crosslinked micelle	High GSH	Doxorubicin	Bladder cancer cell line (T24)	*In vitro*	Enhanced cellular uptake; IC50 reduced by 72%	(39)
Lipid PPBC LNPs	NIR light	Photosensitizer	Orthotopic bladder cancer (mouse)	Preclinical (*in vivo*)	PA/FL imaging-guided PTT/PD T; 70% tumor growth inhibition	(7)
Chitosan-siRNA nanocomple x	Mucosal adhesion	siNrf2 + rapamycin	Bladder cancer organoid	Ex vivo	Silenced Nrf2; restored cisplatin sensitivity	(4, 43)

### 2.3 Strategies for nanoparticles in overcoming drug resistance in bladder cancer treatment

#### 2.3.1 Evading drug efflux pump mechanisms

Nanocarriers utilize endocytosis to bypass efflux pumps such as P-gp, representing a key approach to overcoming bladder cancer cell resistance. In bladder cancer cells, efflux pumps such as P-gp (P-glycoprotein) actively pump chemotherapy drugs out of the cell, thereby significantly reducing intracellular drug concentrations. This leads to diminished therapeutic efficacy and the development of resistance. Conventional drugs struggle to penetrate this mechanism, but nanocarriers can enter cells via endocytosis, preventing drug exposure to efflux pumps and thereby increasing intracellular drug concentration. This mechanism not only enhances drug bioavailability but also increases its cytotoxicity against drug-resistant cells. A nanomedicine system that utilizes macrophages to mimic nanovesicles carrying cisplatin and small interfering RNA (siRNA) targeting the ERCC2 gene achieves highly efficient intracellular delivery. This approach significantly increases cisplatin accumulation in bladder cancer cells, suppresses ERCC2 gene expression, enhances chemotherapy sensitivity, promotes cancer cell apoptosis, and effectively inhibits tumor growth (10). Additionally, targeted and sustained-release nanocarriers can be engineered to improve drug selectivity toward tumor cells while reducing toxic side effects on normal tissues. To address drug resistance, nanocarriers not only evade efflux pump-mediated drug clearance but also synergistically enhance drug efficacy through multiple mechanisms. When targeted self-assembling prodrug nanocarriers are internalized by tumor cells, the acidic lysosomal environment triggers prodrug release and disrupts lysosomal membrane integrity. This prevents drug sequestration and degradation within lysosomes, significantly reducing the half-maximal inhibitory concentration (IC_50_) and effectively overcoming drug tolerance (38). This strategy not only elevates intracellular drug concentrations but also disrupts intracellular drug resistance pathways, enhancing the cytotoxic potency of chemotherapeutic agents.

In summary, smart responsive nanoplatforms circumvent traditional drug efflux pumps via endocytosis, markedly increasing intracellular drug concentrations in bladder cancer cells. This approach enhances drug sensitivity and cytotoxicity in resistant cells, representing a promising technical approach to overcoming therapeutic resistance in bladder cancer. Future designs integrating gene silencing, small-molecule drugs, and immunomodulators into nanocarriers could enhance therapeutic efficacy, achieving precise and efficient bladder cancer treatment (37).

#### 2.3.2 Targeted delivery of drug resistance-related gene inhibitors

Drug resistance during bladder cancer treatment severely impacts clinical outcomes. Inhibiting resistance-related genes aims to overcome therapeutic barriers. Delivering small interfering RNA (siRNA) or microRNA (miRNA) via nanoparticles to suppress the expression of drug resistance genes has proven to be an effective and promising therapeutic approach. Specifically, overexpression of the drug resistance gene Bcl-2 enhances tumor cells’ resistance to apoptosis, reducing their sensitivity to chemotherapy drugs. Delivering siRNA or miRNA via nanoparticle carriers enables specific, selective regulation of drug resistance gene expression levels, thereby restoring tumor cell sensitivity to drugs.

Abnormal activation of the mTOR signaling pathway promotes tumor growth and resistance in bladder cancer. Bcl-2, another anti-apoptotic protein, is regulated by this pathway. Encapsulating rapamycin within poly (lactic-co-glycolic acid) (PLGA) nanoparticles for targeted delivery not only enhances rapamycin’s water solubility and bioavailability but also significantly reduces the expression of drug-resistance-related genes, such as mTOR and Bcl-2, in bladder cancer cells, thereby increasing apoptosis rates and drug sensitivity (48). This strategy integrates antitumor drug activity with genetic regulatory mechanisms, offering novel prospects for nanoparticle-mediated delivery of gene inhibitors.

Additionally, siRNA delivery targeting the transcription factor Nrf2 can overcome cisplatin resistance in bladder cancer. Nrf2, a key regulator of antioxidant and cytoprotective gene expression, exhibits elevated expression correlated with cisplatin resistance in bladder cancer cells. By loading siNrf2 onto guanidino-terminated carboxysilane dendritic polymer (GCD) nanocarriers, Nrf2 gene expression can be specifically suppressed, significantly enhancing the sensitivity of cisplatin-resistant bladder cancer cells to cisplatin while maintaining a favorable safety profile (43). This nanodelivery system not only enhances siRNA stability and cellular uptake efficiency but also effectively reduces non-specific toxicity, representing a breakthrough in gene therapy.

Nanotechnology enables the precise delivery of inhibitors of drug resistance genes. By optimizing particle size, surface modification, and release mechanisms, drug accumulation and retention time within bladder tumors can be increased, thereby overcoming the bladder mucosal barrier and enhancing therapeutic efficacy (49). In summary, siRNA or miRNA delivery systems utilizing nanocarriers can accurately and efficiently suppress drug resistance-associated genes such as Bcl-2 and Nrf2, reduce the expression of resistance genes, and restore drug sensitivity. This provides novel therapeutic approaches and insights for combating chemotherapy resistance in bladder cancer. In the future, multifunctional nanoplatforms and targeted delivery technologies will advance translational research to enable precision treatment and reverse resistance in bladder cancer.

#### 2.3.3 Modulating the tumor microenvironment

The tumor microenvironment (TME) plays a pivotal role in the progression and treatment resistance of bladder cancer. Characterized by tumor hypoxia, high reductive stress, immune suppression, and abnormal extracellular matrix, the TME promotes malignant transformation and significantly impedes therapeutic outcomes. Intelligent, responsive, multifunctional nanoplatforms that precisely modulate the TME can enhance drug delivery efficacy, accelerate therapeutic response, and overcome drug resistance, representing a primary direction for comprehensive bladder cancer treatment. Specific regulatory mechanisms include: utilizing physical therapies to alleviate tumor hypoxia; depleting intracellular glutathione (GSH) to disrupt redox balance; and altering the immune microenvironment to promote antitumor immune responses.

First, physical methods such as photothermal therapy (PTT) effectively reduce tumor hypoxia, thereby enhancing the efficacy of chemotherapy and photodynamic therapy (PDT). Tumor hypoxia not only reduces drug activity but also promotes drug resistance. Under near-infrared light irradiation, heat generated by photothermal materials on the nanoplatform stimulates local tumor blood flow, increasing oxygen delivery and improving blood flow. Nanoplatforms containing MnO_2_ catalyze hydrogen peroxide within tumors to generate oxygen, elevating local oxygen partial pressure, mitigating hypoxia, activating autophagy inhibitors, and enhancing radiotherapy sensitivity (50). Additionally, nanoplatforms incorporating photothermal agents and oxygen-carrying materials have been designed for bladder cancer, utilizing photothermal effects to increase oxygen levels in tumor tissues, thereby boosting drug efficacy and immune therapy responses (39, 51).

Secondly, high intracellular glutathione (GSH) levels constitute a critical component of antioxidant defense, maintaining redox homeostasis in tumor cells and suppressing ROS-induced cytotoxicity. Utilizing smart nanoplatforms to deplete GSH disrupts tumor redox balance, enhancing the efficacy of chemotherapy and photodynamic therapy (PDT). The Fe/Cu bimetallic nanozyme system effectively depletes tumor GSH. When combined with glucose oxidase (GOx), it generates hydrogen peroxide, which catalyzes the Fenton reaction and produces abundant ROS. This induces ferroptosis and cupric-mediated cell death in tumor cells, enhances immunogenic cell death (ICD), promotes dendritic cell maturation, and facilitates T-cell infiltration—thereby reconstructing the tumor immune microenvironment (40). Similarly, nanoplatforms loaded with photosensitizers and redox-regulating agents (e.g., SPS@ZnO_2_NPs) release hydrogen peroxide and deplete GSH, enhancing PDT efficacy while promoting tumor cell apoptosis and immune activation (52). Other nanoplatform designs simultaneously modulate tumor microenvironment antioxidant activity and drug release, achieving synergistic effects between chemotherapy and immunotherapy (53).

Finally, the immunosuppressive nature of the tumor microenvironment significantly enhances tumor responsiveness to immunotherapy. Smart nanoplatforms that modulate the immune microenvironment to promote antitumor immune responses represent a critical research direction.

Multifunctional nanoplatforms can promote polarization of tumor-associated macrophages toward the M1 phenotype, activate dendritic cell maturation, enhance cytotoxic T lymphocyte infiltration, and suppress proliferation of immunosuppressive cell populations (regulatory T cells, myeloid suppressor cells, etc.), thereby reversing immune suppression. A MoS_2_ nanoplatform modified with M1 macrophage-derived exosomes utilizes photothermal effects to generate reactive oxygen species (ROS), activating the immune system to promote CD8^+^ T cell infiltration and downregulate PD-L1 expression, significantly inhibiting bladder tumor growth (8). Additionally, a nuclear-targeting nanoplatform combining photodynamic therapy with chemotherapy activates the cGAS-STING pathway, enhancing antitumor immune responses, promoting dendritic cell maturation and memory T cell production, reshaping the immune microenvironment, and markedly inhibiting bladder cancer (9). Nanocarriers can synergize with immune checkpoint inhibitors and metabolic modulators to reduce levels of immunosuppressive molecules (e.g., CD73, PD-L1) in the tumor microenvironment while promoting cytokine production, thereby enhancing immunotherapy efficacy (11, 54). Furthermore, integrating the CRISPR-Cas13a system with PD-L1 expression via nanoplatforms and modulating the immune microenvironment with chemotherapeutic agents achieves highly efficient immune activation, thereby treating bladder cancer (55).

In summary, smart responsive multifunctional nanoplatforms achieve multiple objectives by regulating the tumor microenvironment: reducing hypoxia, depleting GSH to disrupt redox homeostasis, and reshaping the immune microenvironment. This not only enhances the efficacy of chemotherapy and photodynamic therapy for bladder cancer but also significantly improves patient responsiveness to immunotherapy.

**Figure 6:**
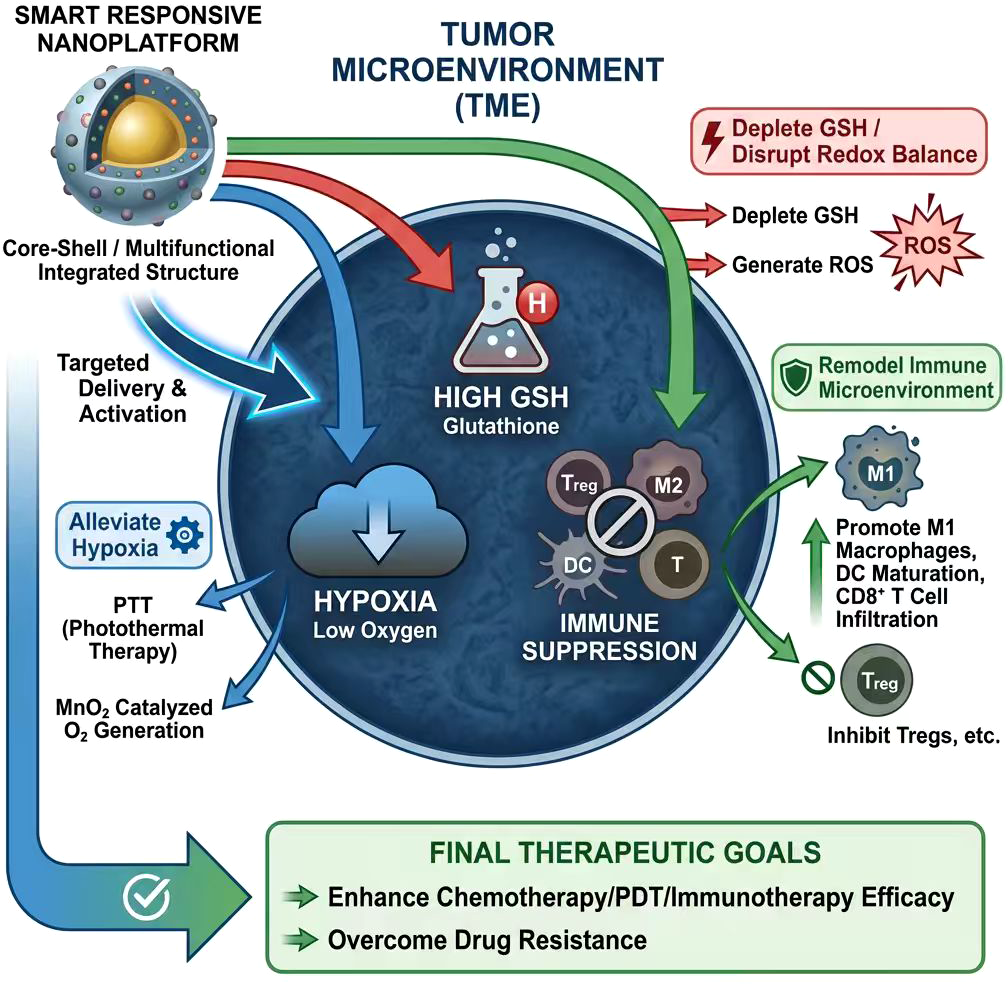
Schematic diagram of tumor microenvironment regulation strategies.

Integrating physical therapy, chemotherapy, and immune modulation through these nanoplatforms enables precision treatment and counteracts drug resistance in bladder cancer. With ongoing advancements in nanomaterial design and bio-responsive mechanisms, multifunctional nanoplatforms hold promise for integrated bladder cancer diagnosis and treatment in clinical applications, offering patients safer and more efficient therapeutic approaches (**Figure 6**).

### 2.4 From basic research to clinical practice: a translational medicine perspective

#### 2.4.1 Preclinical research advances

Smart, responsive multifunctional nanoplatforms have demonstrated significant therapeutic potential in preclinical bladder cancer studies, with multiple nanotechnologies achieving promising outcomes in mouse models. Due to bladder cancer’s high recurrence rate and the highly impermeable barrier formed by urothelium, traditional drugs administered via intravesical instillation struggle to reach tumor tissues, compromising both therapeutic efficacy and diagnostic accuracy. Addressing this challenge, nanotechnology development offers a novel pathway to enhance bladder cancer treatment. Nanoparticles can penetrate the uroepithelial barrier, enabling precise targeting within drug delivery systems. Surface functionalization facilitates active recognition and binding to tumor cells, significantly improving treatment specificity and efficacy (56).

Diagnosis-treatment integrated nanoplatforms combine tumor targeting, real-time imaging, and combined therapy, demonstrating promising applications in preclinical models. Beyond drug loading, these nanoparticles integrate multimodal imaging probes—such as fluorescent labels and magnetic resonance imaging (MRI) contrast agents—enabling more precise and dynamic tumor localization and monitoring. Real-time tumor monitoring enables adjustments to treatment plans, enhancing personalized, precise therapeutic approaches. Additionally, synergistic combination therapies—such as chemotherapy paired with photodynamic therapy—delivered via nanoplatforms significantly boost antitumor activity while reducing treatment-related toxicity (56).

Specific preclinical findings demonstrate that these nanoplatforms not only prolong survival but also effectively suppress tumor growth and recurrence in mouse bladder cancer models. Multifunctional nanoparticles enhance drug accumulation within the tumor microenvironment while minimizing damage to normal tissues. Imaging technologies provide precise margins for surgical resection, maximizing tumor removal while preserving healthy tissue and improving patient prognosis (56).

Thus, smart-responsive multifunctional nanoplatforms show great promise in preclinical bladder cancer research. Through the synergistic integration of tumor targeting, real-time imaging, and combined therapy, these nanotechnologies have overcome limitations of traditional bladder cancer treatments while laying a solid foundation for future clinical applications. Future research should further optimize nanoparticle biocompatibility and functional combinations to facilitate broader adoption in precision diagnosis and treatment of bladder cancer.

#### 2.4.2 Challenges in clinical translation

As intelligent, multifunctional nanoplatforms advance rapidly in bladder cancer diagnostics and therapy, clinical translation faces numerous complex challenges. These primarily involve biosafety, large-scale production, and regulatory approval—factors that determine whether nanoplatforms can successfully enter clinical application. Addressing these issues is crucial for advancing the widespread use of nanotechnology in bladder cancer management.

##### 2.4.2.1 Biosafety concerns

The biosafety of nanomaterials is paramount for clinical translation, as their long-term toxicity, immunogenicity, and metabolic pathways within the body remain poorly understood. While nanomedicines offer distinct therapeutic advantages, their complex material composition and nanoscale characteristics may trigger unforeseen biological responses.

Some nanoplatforms can induce immune activation through surface chemistry or particle size, leading to inflammatory responses or immune rejection. Immunogenicity thus demands rigorous evaluation for clinical use. More critically, significant uncertainties remain regarding the *in vivo* metabolism and clearance mechanisms of nanomaterials. Prolonged retention in the body and accumulation in specific organs may lead to potential toxicity. Additionally, the safety of nanoparticle degradation products and their potential to damage detoxification organs, such as the liver and kidneys, are key indicators for assessing biosafety. Existing research has employed microfluidic assembly techniques to enhance the biocompatibility and stability of lipid nanoparticles, demonstrating promising clinical translation potential. However, systematic toxicological studies remain insufficient (6, 7). Therefore, future efforts should integrate multi-level *in vivo* and *in vitro* safety evaluation systems to comprehensively understand the complex interactions between nanomaterials and biological systems, ensuring their safe and effective clinical application.

##### 2.4.2.2 Challenges in large-scale production

Industrial-scale production of nanomedicines faces multifaceted challenges, including reproducibility, quality control, and cost-effectiveness. The preparation processes for nanomaterials are complex, where even minor variations in process parameters can significantly alter the physicochemical properties, drug loading efficiency, and release behavior of the final product, thereby impacting therapeutic efficacy and safety. Taking lipid nanoparticles and polymeric nanoparticles as examples, while microfluidic assembly and self-assembly techniques can enhance preparation uniformity, scaling up production still presents challenges in equipment adaptability and process stability. Furthermore, quality control standards for nanomedicines remain inconsistent. Critical metrics, such as particle size distribution, surface charge, drug encapsulation efficiency, and release kinetics, require rigorous, comprehensive testing protocols.

##### 2.4.2.3 Regulatory approval barriers

Smart-responsive multifunctional nanoplatforms represent novel composite nanotherapeutic approaches that pose significant regulatory challenges. While approval processes for single-component drugs are relatively mature, nanoplatforms typically constitute complex combination products comprising drug carriers, targeting molecules, and diagnostic/therapeutic functions. Evaluation standards for their safety and efficacy remain unclear, leading to complex, protracted approval procedures. Furthermore, regulatory agencies worldwide differ in their definitions, classifications, and testing methods for nanomaterials, and there are no unified international standards. This disparity increases the workload for multinational clinical trials and commercialization efforts. The mechanisms of action and *in vivo* behavior of nanomedicines are complex and multimodal. Conventional pharmacokinetic and toxicological evaluation metrics inadequately reflect their characteristics, prompting regulatory bodies to demand additional nonclinical and clinical data to support them, thereby increasing R&D costs. Meanwhile, nanomedicine platform designs evolve rapidly, yet regulatory policies struggle to keep pace, leading to lagging approval processes that hinder swift market entry for novel therapies. Consequently, the industry advocates establishing specialized review guidelines and standardized methods for nanomedicines, promoting multi-faceted collaboration among academia, industry, and regulators. A scientifically sound, standardized regulatory framework is needed to advance the clinical application of nanotechnology (6, 57). Only by overcoming regulatory barriers can smart responsive nanoplatforms truly transition from the laboratory to clinical practice.

### 2.5 Future development directions for smart responsive nanoplatforms

#### 2.5.1 Developing more precise and biocompatible novel nanomaterials

Nanomaterials are increasingly applied in bladder cancer diagnosis and treatment, with their performance directly impacting therapeutic outcomes. To achieve more precise targeted therapy and reduce adverse biological reactions, developing highly biocompatible and safe nanomaterials is a current research priority. Biomembrane-coated nanoparticles can effectively enhance biocompatibility while improving targeting efficiency. Specifically, biomembrane coatings provide natural camouflage, enabling nanoparticles to evade immune system recognition and clearance. This prolongs drug circulation time *in vivo* and enhances targeted drug accumulation. Platelet-coated nanocarriers leverage platelet surface molecules, such as p-selectin, to achieve precise recognition and targeted delivery to bladder tumors, inducing tumor cell pyroptosis and enhancing immunotherapy efficacy (58). Additionally, nanomaterial design must consider degradability and safety. Ideal nanomaterials should exhibit favorable degradability, metabolizing into non-toxic substances within the body to prevent potential toxicity from prolonged accumulation. Various polymeric materials—including chitosan, hyaluronic acid, and poly (lactic-co-glycolic acid) (PLGA)—have been extensively studied and employed as nanocarriers. These materials exhibit excellent biocompatibility and allow controlled degradation by adjusting molecular weight and structure (59). Furthermore, metal-based nanomaterials, such as zinc oxide (ZnO) and its compounds, exhibit significantly reduced toxicity when coated with natural biomolecules (e.g., chitosan, alginate), while retaining strong antimicrobial activity and therapeutic value (60). Additionally, multifunctional diagnostic and therapeutic platforms based on carbon-based nanomaterials (e.g., carbon dots, nanodiamonds, graphene oxide) show great developmental potential (61–63). Overall, the rational design combining biofilm coating technology with polymeric, metallic, or carbon-based nanomaterials not only achieves efficient targeted delivery to bladder cancer cells but also markedly enhances the biocompatibility and safety of nanomaterials, providing a solid foundation for precision treatment of bladder cancer.

The advantages of biofilm-coated nanoparticles are primarily as follows. First, the complex lipid and protein components of natural biological membranes confer excellent immune-evasion capabilities to nanoparticles, thereby reducing clearance by the mononuclear phagocyte system. This extends circulation time and enhances accumulation in tumor tissues.

Second, specific receptors and adhesion molecules on cell membranes enable recognition of the tumor microenvironment or specific tumor markers, thereby improving nanoparticle targeting and minimizing side effects on healthy tissues. Using nanoparticles coated with tumor cell membranes achieves autotargeting, improving treatment accuracy (64). Third, the biofilm coating enhances nanoparticle stability, preventing aggregation or premature drug release in the circulatory system, thereby ensuring the efficacy of the drug delivery system. Finally, the preparation process for biofilm-coated nanoparticles is increasingly mature, offering good controllability and scalability, which facilitates clinical translation and industrialization.

In material design, safety and degradability are equally critical. Studies indicate that non-degradable nanomaterials can induce long-term toxicity and immune responses, limiting their clinical application. Therefore, nanoparticles made from biodegradable polymers (e.g., PLGA, chitosan) gradually decompose into harmless metabolites after drug delivery, reducing the burden on the organism (59, 65). Additionally, highly toxic elements must be avoided in the design of nanomaterials. For instance, while cadmium-based quantum dots exhibit excellent optical properties, their potential toxicity limits widespread use. Low-toxicity semiconductor quantum dots (silver, copper, zinc, etc.) are increasingly becoming research hotspots as alternatives (66). During the functionalization of nanomaterials, incorporating molecules with diverse biological functions—such as proteins, polysaccharides, and surface stabilizers—enhances nanoparticle targeting and stability while significantly reducing immunogenicity, thereby improving overall biosafety (67, 68). Furthermore, recent advancements in artificial intelligence and deep learning technologies have opened new avenues for rapidly designing highly efficient, biocompatible nanomaterials. These approaches enable molecular-level prediction of nanomaterial toxicity and cellular uptake efficiency, facilitating precision design and high-throughput screening (69).

Therefore, the future development of intelligent, multifunctional nanoplatforms for bladder cancer should fully leverage the advantages of biofilm-coating technology and biodegradable polymers. By integrating low-toxicity inorganic nanomaterials with functionalization strategies, we can design nanocarrier systems featuring excellent biocompatibility, precise targeting, and safe, controllable degradation. This will provide robust technical support for early bladder cancer diagnosis, overcoming treatment resistance, and personalized therapy. Concurrently, biosafety assessments and clinical translation studies of relevant nanomaterials require reinforcement to ensure their safety and efficacy in clinical applications.

#### 2.5.2 Exploration of personalized nanomedicine

With the advancement of precision medicine, developing personalized nanotherapeutic regimens based on a patient’s tumor molecular profiling represents a frontier in nanomedicine research. Traditional treatments often yield suboptimal outcomes due to tumor molecular heterogeneity and patient variability. Nanotechnology, leveraging its high tunability, enables precise recognition of tumor cells and targeted drug delivery, thereby enhancing therapeutic efficacy and safety (70). Specifically, personalized nanomedicine relies on in-depth analysis of a patient’s genomic and proteomic data to identify key driver genes, signaling pathways, and protein expression profiles. This enables the design of nanocarriers capable of intervening at these molecular targets.

Nanoparticles surface-modified with specific ligands can leverage receptor-mediated endocytosis to achieve effective tumor cell recognition and drug release, thereby avoiding damage to healthy tissues and reducing systemic toxicity (6).

Additionally, leveraging genomics and proteomics to optimize drug combinations represents a crucial approach for enhancing nanotherapeutic efficacy. Integrating multi-omics data reveals mechanisms of tumor multidrug resistance and signaling pathways, enabling the design of multidrug nanocarriers that achieve synergistic effects. Nanoplatforms can load multiple components, such as chemotherapeutic agents, gene-editing tools, or immunomodulators, attacking tumor cells and their microenvironment from various angles to overcome the limitations and resistance associated with monotherapy (71). Smart responsive nanostructures can control targeted drug release based on tumor microenvironment characteristics—including pH, reducing agent content, and enzyme activity—achieving high local concentrations at the tumor site to enhance treatment accuracy and safety (72).

Notably, personalized nanomedicine relies on integrating technologies such as high-throughput sequencing and machine learning. Big data analysis enables dynamic treatment plan adjustments and efficacy predictions, ultimately achieving true precision medicine (73). Despite these advancements, challenges remain in biocompatibility, immune responses, and scalable production, necessitating multidisciplinary collaboration to advance clinical applications. Overall, developing personalized nanotherapeutic regimens based on a patient’s tumor molecular profile and optimizing drug combinations using multi-omics data represent future trends in cancer treatment. This approach holds significant potential to enhance therapeutic efficacy, reduce adverse effects, and overcome treatment resistance (74, 75).

#### 2.5.3 AI-assisted nanodrug design and treatment planning

With the advancement of nanomedicine, rationally designing nanoparticles to achieve high-efficiency treatment and overcome therapeutic resistance has become a research hotspot. The application of artificial intelligence (AI) technology has opened new pathways and provided insights into nanomedicine design and treatment planning. AI can process complex multidimensional data and rapidly screen and predict nanoparticle properties, thereby improving drug carrier design, enhancing therapeutic efficacy, and enabling precision medicine.

First, using AI to predict nanoparticle performance and therapeutic outcomes represents one direction in the design of intelligent nanomedicine. Traditional nanomedicine design largely relies on extensive experimentation and trial-and-error, which is time-consuming, labor-intensive, and inefficient. Leveraging machine learning and deep learning algorithms, AI uncovers structure-function relationships within large datasets and accurately predicts key parameters, including nanoparticle pharmacokinetics, biodistribution, cellular uptake, and immune responses. Combining high-throughput screening with AI models enables rapid identification of optimal nanomaterial combinations and surface modifications, significantly enhancing the targeting and biocompatibility of nanomedicines (76, 77). Furthermore, AI can simulate complex biological processes, such as protein crown formation, to understand the *in vivo* dynamics of nanoparticles, thereby predicting therapeutic efficacy and potential toxicity (78).

Second, AI enables intelligent optimization of precision treatment regimens. Nanodrugs typically involve multi-drug combination delivery, where synergistic effects are influenced by dosage and administration timing. Due to interpatient variability, fixed-dose regimens rarely achieve optimal efficacy. AI integrates clinical, genomic, and drug response data to dynamically adjust nanodrug dosages and administration schedules, achieving personalized treatment goals (79, 80). Machine learning models can predict patient responses to different nanodrug combinations, enabling clinicians to select optimal treatment regimens that enhance therapeutic success and patient comfort (81). Furthermore, AI leverages automated experimental platforms to accelerate nanodrug development, shorten R&D cycles, and improve translational efficiency (82).

For bladder cancer diagnosis and treatment, AI-assisted design and formulation of nanomedicines, alongside therapeutic planning, are of significant importance. Early diagnosis and treatment resistance in bladder cancer present complex and variable challenges that traditional approaches cannot adequately address. AI-driven nanoplatforms enable precise modulation of nanoparticle surface properties, drug loading capacity, and release kinetics, thereby enhancing tumor-targeted delivery efficiency and therapeutic efficacy. AI can also integrate with nanomedicine’s diagnostic capabilities to enable a seamless transition between treatment and monitoring, thereby advancing the integrated bladder cancer therapy process (83, 84).

In summary, artificial intelligence technology empowers nanomedicine design and treatment planning, transforming it from an empirical to a data-driven field. As data standardization, model transparency, and regulatory frameworks mature, AI-assisted nanomedicine design will further advance precision treatment for diseases like bladder cancer, improving patient quality of life (85, 86).

#### 2.5.4 Advancing clinical trial design and implementation

Evaluating the safety and efficacy of intelligent, responsive, multifunctional nanoplatforms for bladder cancer treatment through clinical trials is crucial for assessing their practical applicability. Given the complex nature and multifunctionality of these platforms, clinical trial designs must fully account for their pharmacokinetics, immunomodulatory effects, and tumor-targeting properties. The nanotherapeutic system combining M1 macrophage-derived exosomes with molybdenum disulfide nanoparticles (M1 EVs@MoS_2_) demonstrated promising tumor suppression in preliminary animal studies, alongside enhanced CD8+ T cell infiltration, dendritic cell activation, and reduced PD-L1 expression (8). Clinical trials for such platforms should be designed as multi-stage, dose-escalation studies primarily monitoring changes in immune activation markers and tumor burden, while evaluating potential immune-related adverse reactions. Additionally, for combination therapies pairing nanomedicines with immune checkpoint inhibitors, a control group distinguishing single-agent versus combination effects could utilize macrophage-derived nanovesicles carrying CD73 inhibitors alongside anti-PD-L1 antibody immunotherapy. This approach has demonstrated favorable safety profiles and superior antitumor activity (11). Clinical trials also require multimodal imaging techniques (e.g., photoacoustic and fluorescence imaging) to enable real-time tracking of nanomedicines and to evaluate efficacy, thereby enhancing trial accuracy and scientific rigor (7).

Strengthening multicenter collaboration is essential to advancing the clinical translation of nanomedicine. Given the heterogeneity of bladder cancer patient populations and diverse treatment needs, synchronized clinical trials across different regions and medical institutions can collect broader clinical data to enhance the external validity and generalizability of research. Multicenter collaboration facilitates resource sharing and accelerates the transition of nanoplatforms from laboratory to clinical settings. A nanoplatform utilizing bioengineered bacterial outer membrane vesicles (OMVs) as carriers for delivering fusion peptides has demonstrated anti-bladder cancer efficacy and excellent biocompatibility across multiple models (87–89). Integrating this platform with multicenter patient resources could expedite related clinical studies. Additionally, multicenter clinical studies can address technical challenges such as scaling nanomedicine production and quality control, enhance trial standardization, and provide robust scientific evidence for clinical application of nanoplatforms (6).

Future trial designs should incorporate patient individualization and nanoplatform smart response mechanisms, integrating precision medicine principles to optimize treatment strategies. Utilizing the CRISPR-Cas13a nanoplatform to regulate PD-L1 expression represents a novel therapeutic approach that combines molecular targeting with immune modulation (55). Incorporating biomarker detection and immune-related parameter evaluation into clinical trials enables efficacy prediction and early detection of adverse reactions, thereby enhancing treatment safety and effectiveness.

## 3 Conclusion

Intelligent, responsive, multifunctional nanoplatforms have rapidly emerged in the diagnosis and treatment of bladder cancer, heralding a new era in tumor therapy. As a seasoned review expert in the medical field, one cannot overlook their role in medical advancement and the potential challenges they present. From an expert perspective, these smart nanoplatforms demonstrate strong adaptability and efficacy in responding to the tumor microenvironment and external stimuli, effectively addressing the drawbacks of traditional treatments, which are prone to drug resistance. This not only enriches bladder cancer treatment options but also provides a practical technical pathway toward integrated diagnosis and therapy.The design philosophy of multimodal nanoparticles is a key driver of progress in this field. By enhancing drug targeting and controlled release, these platforms achieve the aforementioned goals while minimizing damage to healthy tissues—all while ensuring optimal drug concentration and duration of action. Additionally, the nanoplatform enables real-time monitoring during treatment, enhancing safety and controllability while providing scientific grounds for personalized therapeutic strategies. This “diagnosis-treatment integration” model, characterized by combined diagnostic and therapeutic functions, aligns with precision medicine trends and offers bladder cancer patients greater therapeutic opportunities.

Nevertheless, the gap between current research achievements and clinical application must be viewed rationally. Biosafety concerns remain the primary barrier to widespread adoption of smart nanoplatforms. Research on the *in vivo* metabolic pathways, potential toxicity, and long-term safety of nanomaterials remains insufficient. Furthermore, scaling production and quality control from laboratory to clinical settings presents dual technical and economic challenges. The complexity and lack of unified standards in regulatory approval processes add further uncertainties to the clinical translation of nanoplatforms. Therefore, future research should not focus solely on technological innovation but also on strengthening interdisciplinary collaboration, refining safety evaluation systems, and actively promoting standardized production processes compliant with regulatory requirements.

Looking ahead, the development of smart, responsive, multifunctional nanoplatforms must prioritize material safety and functional enhancement. Leveraging artificial intelligence technologies enables accurate prediction and control of nanoparticle behavior, thereby improving treatment personalization and dynamic adjustability. Furthermore, integrating personalized medicine concepts—tailoring nanotherapies based on patient genomics and metabolomics—can enhance treatment efficacy while reducing side effects. Cross-disciplinary integration of emerging technologies and multidisciplinary knowledge represents a pathway to advancing the clinical translation of smart nanoplatforms. In summary, smart, responsive, multifunctional nanoplatforms offer novel approaches for the precision treatment of bladder cancer. While acknowledging its revolutionary significance, the scientific community should rationally recognize existing limitations and challenges. By continuously refining nanomaterial design, strengthening safety evaluations, optimizing production processes, and actively integrating artificial intelligence with personalized medicine, this field holds promise for a successful transition from laboratory research to clinical application. This will ultimately achieve precise, efficient, and low-toxicity bladder cancer treatment, benefiting more patients.
